# Rubidium and potassium levels are altered in Alzheimer’s disease brain and blood but not in cerebrospinal fluid

**DOI:** 10.1186/s40478-016-0390-8

**Published:** 2016-11-14

**Authors:** Blaine R. Roberts, James D. Doecke, Alan Rembach, L. Fernanda Yévenes, Christopher J. Fowler, Catriona A. McLean, Monica Lind, Irene Volitakis, Colin L. Masters, Ashley I. Bush, Dominic J. Hare, the AIBL research group

**Affiliations:** 1 The Florey Institute of Neuroscience, The University of Melbourne, Parkville, VIC Australia; 2 Cooperative Research Centre for Mental Health, Parkville, VIC Australia; 3 The Australian e-Health Research Centre, Herston, QLD Australia; 4 CSIRO Preventative Health Flagship, Molecular Science and Engineering, Parkville, VIC Australia; 5 Department of Anatomical Pathology, The Alfred Hospital, Melbourne, VIC Australia; 6 Department of Medicine, Central Clinical School, Monash University, Clayton, VIC Australia; 7 Elemental Bio-imaging Facility, University of Technology, Broadway, Sydney, NSW Australia; 8 https://aibl.csiro.au/about/aibl-research-team

## Abstract

Loss of intracellular compartmentalization of potassium is a biochemical feature of Alzheimer’s disease indicating a loss of membrane integrity and mitochondrial dysfunction. We examined potassium and rubidium (a biological proxy for potassium) in brain tissue, blood fractions and cerebrospinal fluid from Alzheimer’s disease and healthy control subjects to investigate the diagnostic potential of these two metal ions. We found that both potassium and rubidium levels were significantly decreased across all intracellular compartments in the Alzheimer’s disease brain. Serum from over 1000 participants in the Australian Imaging, Biomarkers and Lifestyle Flagship Study of Ageing (AIBL), showed minor changes according to disease state. Potassium and rubidium levels in erythrocytes and cerebrospinal fluid were not significantly different according to disease state, and rubidium was slightly decreased in Alzheimer’s disease patients compared to healthy controls. Our data provides evidence that contrasts the hypothesized disruption of the blood-brain barrier in Alzheimer’s disease, with the systemic decrease in cortical potassium and rubidium levels suggesting influx of ions from the blood is minimal and that the observed changes are more likely indicative of an internal energy crisis within the brain. These findings may be the basis for potential diagnostic imaging studies using radioactive potassium and rubidium tracers.

**Electronic supplementary material:**

The online version of this article (doi:10.1186/s40478-016-0390-8) contains supplementary material, which is available to authorized users.

## Introduction

Mitochondrial dysfunction and impaired energy metabolism are features that immediately precede neuronal loss in Alzheimer’s disease (AD) [1]. Oxidative stress arising from neurotoxic β-amyloid (Aβ) accumulation and oligomerization causes a loss in membrane integrity in the synapse [2], which is heavily dependent on sufficient ATP production to regulate ion transport in and out of the cell [3]. Oligomeric Aβ species adversely affect cellular function through a range of hypothesized mechanisms, a number of which directly compromise both energy production and membrane potential [4, 5].

Impaired energy production related to AD pathology has been of research interest. Positron emission tomography (PET) has identified a metabolic decrease in glucose consumption in the AD brain [6], which is indicative of reduced neural activity [7] and direct impairment of Na^+^/K^+^-ATPase-regulated intramembrane ion transport [8]. Thus, there is potential that markers of modified K^+^ handling may be indicative of AD, and could be a useful preclinical marker of increased AD risk.

As a group 1 alkali metal, rubidium (Rb^+^) has similar biochemical characteristics to K^+^ [9]. Though it has no known biological function, Rb^+^ is present in almost all biological systems due to its ability to readily exchange with K^+^ [10]. Assessment of Rb^+^ is a useful proxy for K^+^, so much so that turnover of radioactive ^86^Rb^+^ has been successfully used as a measure of basal metabolic rate [11]. Rubidium assays are also less prone to environmental contamination, and the ion is present in biological matrices at concentrations well suited to contemporary analytical techniques [12].

As is the case with many other biometals, there are numerous conflicting reports of either changes to [13, 14] or stability of [15–17] Rb and K levels in AD, most likely due to low statistical power. Here, we used *post mortem* brain tissue from the Victorian Brain Bank Network along with blood samples from the Australian Imaging, Biomarkers and Lifestyle Flagship Study of Ageing [18], which is one of the world’s largest longitudinal studies of AD, to investigate changes in the regulation of K^+^ and Rb^+^ from a system-wide perspective. We aimed to categorically determine if levels of these metals are altered in AD.

## Methods and materials

### Human brain samples

All brain tissues were obtained from the Victorian Brain Bank Network, and all experiments were approved by the University of Melbourne health sciences, human ethics subcommittee (ID1136882). Tissue was collected at autopsy, frozen at −80 °C, then thawed to −20 °C and sectioned into 1 cm slices. From these, c.a. 5 g samples of frontal cortex were collected and 0.5 g aliquots of grey matter dissected and stored at −80 °C until analysis. Neurological control tissues were defined as free from AD lesions with the number of plaque and tangles were well below the cut-off values for the neuropathological diagnosis of AD (NIA Reagan criteria). No other neurological disease was present.

### Preparation of tissue homogenate fractions

Sample preparation steps for tissue samples were adapted our previously reported methods [19, 20]. Aliquots (0.1–0.3 g) of dissected grey matter from the frontal cortex were thawed on ice and homogenized in single-use BioMasher (Omni International) vials. After centrifugation at 100,000 *g* a 1:4 tissue-to-buffer (w/v) ratio of Tris buffered saline (TBS; 50 mM Tris, pH 8.0, 150 mM NaCl) with EDTA-free protease inhibitors (Roche) was added to each homogenate. Samples were transferred to ultracentrifuge vials, spun at 100,000 *g* for 30 min at 4 °C, and then had the supernatant removed (the ‘soluble’ fraction). The remaining pellet was isolated and resuspended with 1:4 (w/v) 100 mM NaCO_3_ (pH 11.0), then centrifuged again at 100,000 *g* for 30 min at 4 °C to retrieve the ‘vesicular’ fraction. Remaining material was then subjected to extraction of membrane-bound proteins and metals (the ‘membrane’ fraction) by addition of 1:4 (w/v) 7 M urea, 2 M thiourea, 4 % 3-[(3-cholamidopropyl) dimethylammonio]-1-propanesulfonate (CHAPS) and 30 mM Bicine (pH 8.5) and further centrifugation at 100,000 *g* for 30 min at 4 °C. Any remaining ‘insoluble’ material was then digested in 70 % formic acid for c.a. 18 h before centrifugation as performed for all other extractions. No material remained at the conclusion of fractionation experiments.

### Preparation of blood samples

Protocols for the preparation of serum and erythrocytes was followed according to our previously reported method [21]. Whole blood was drawn from AIBL subjects after overnight fasting. For serum collection, blood was drawn into serum-gel 7.5 mL tubes (Sarstedt) and left standing at room temperature for 20 min before centrifugation at 1800 *g* for 15 min. Serum was split into 250 μL aliquots and stored in liquid nitrogen until analysis. After thawing, serum samples were spun briefly at 1800 *g* and diluted 1:10 in 1 % HNO_3_ for analysis. Platelets were recovered by centrifuging a plasma fraction (collected from whole blood in a lithium-heparin tube) at 800 *g* for 15 min and removing the plasma supernatant.

Erythrocytes were collected from blood samples taken after an overnight fast into standard lithium-heparin 7.5 mL tubes (Sarstedt). Samples were spun at 3200 *g* to remove plasma and platelets, leaving erythrocytes that were washed three times in 0.9 % (w/v) NaCl. Erythrocytes were distributed by inverting the tube and then centrifuged at 650 *g* for 10 min, after which the supernatant was removed and samples were spun again at 1500 *g* for 10 min at room temperature. Erythrocytes were then resuspended in 6 mL of 0.1 M PBS and stored in liquid nitrogen until analysis. A 50 μL of thawed erythrocytes were transferred to 1.5 mL polypropylene tubes and digested in equal volumes of 65 % HNO_3_ and ≥30 % H_2_O_2_ on a hotplate at 80 °C. After cooling, samples were diluted 1:20 with 1 % HNO_3_. Platelets were prepared for analysis using the same digestion procedure as described for erythrocytes.

### Preparation of CSF samples

Cerebrospinal fluid was collected using the gravity drip method described by Rembach et al. [22]. Patients underwent a lumbar puncture procedure following an overnight fast, and up to 8 mL of CSF was collected directly into a 15 mL polypropylene tube. Samples were centrifuged at 2000 *g* at 4 °C and then divided into 300 μL aliquots and stored on liquid nitrogen. After thawing, 100 μL aliquots were diluted 1:10 with 1 % HNO_3_ for analysis.

### Rubidium and potassium analysis

All metal analyses were performed on an Agilent 7700× Series inductively coupled plasma-mass spectrometer (ICP-MS) using a Teflon MiraMist nebulizer (Burgener Research Inc.) and Scott-type double-pass spray chamber (Glass Expansion). Helium was used as a collision gas to remove potential polyatomic interferences. The instrument was calibrated using multi-element standards (Accustandard, ICP-MS-2-1, ICP-MS-3-1, ICP-MS-4-1; total of 44 elements) containing K and Rb at 0, 5, 10, 50, 100 and 500 μg L^−1^ with ^89^Y introduced online through a T-piece as the internal standard. ^39^K and ^85^Rb were monitored. Seronorm™ L1 and L2 (Sero) were reconstituted in 1:20, 1 % HNO_3_ prior to analysis for use as quality control standards.

### Statistical analysis

All statistical testing was performed in GraphPad Prism v6.0 h using unpaired *t*-tests, one-way ANOVA and Pearson correlation analysis as appropriate. Significance was defined as *p* < 0.05 after Bonferroni *post-hoc* testing.

## Results

### K and Rb levels are consistently decreased in fractionated brain homogenates

**Fig. 1:**
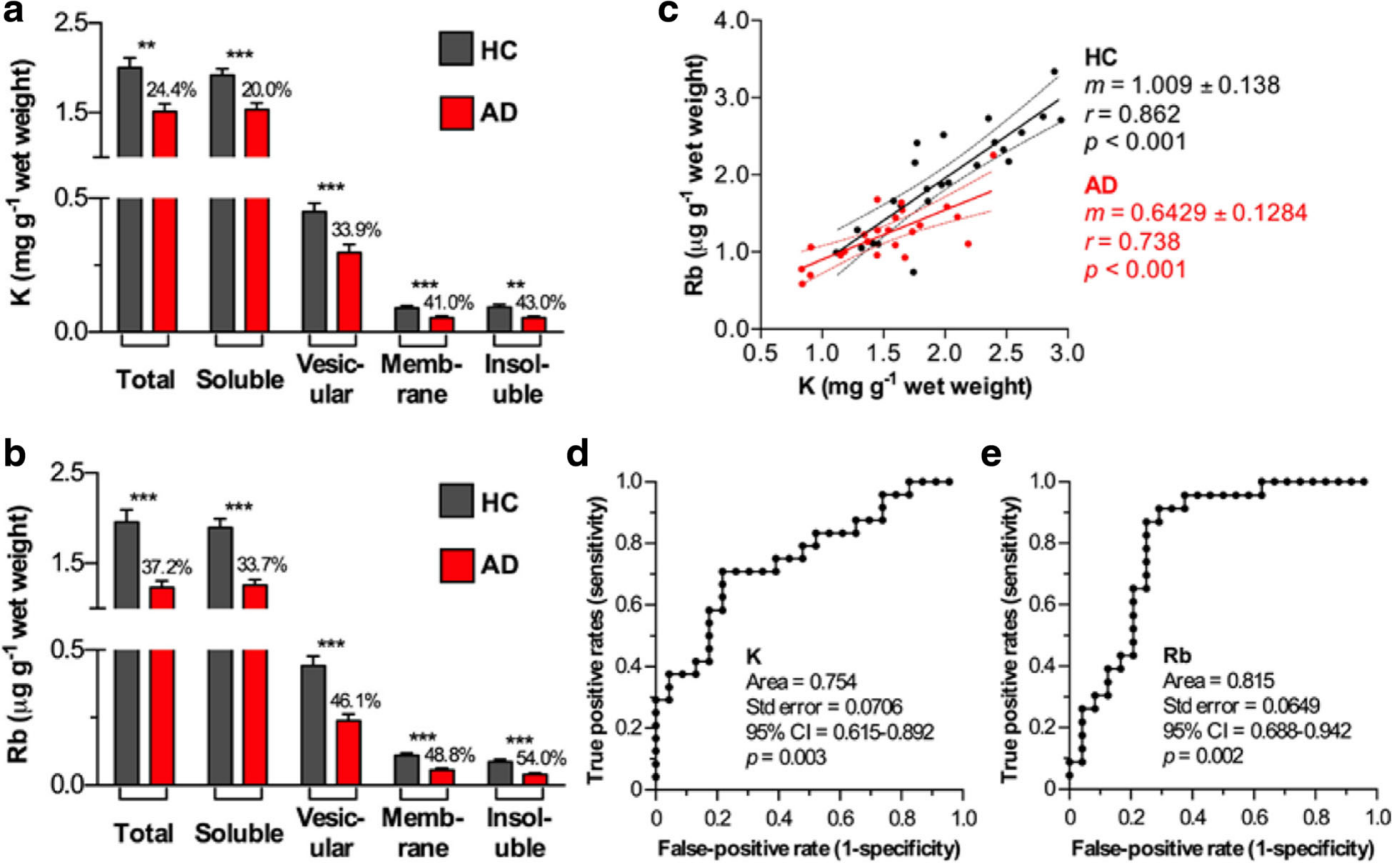
**a**, **b** K and Rb levels in fractionated brain homogenates were consistently decreased in AD brains (*n* = 30 per group; one-way ANOVA; ** *p* < 0.01; *** *p* < 0.001; percentage decrease in AD group compared to healthy controls shown). Error bars represent the standard error of the mean. **c** Both metals showed significant correlation regardless of clinical classification (*p* < 0.001), though the slope of the line of best fit was significantly decreased in the AD group (*p* < 0.05). **d**, **e** Both metals performed well at predicting AD via ROC curve analysis, with Rb slightly superior performance

We analyzed *post mortem* AD and healthy control (HC) frontal cortical tissue (*n* = 30 per group, matched for age and sex; see Table 1 for demographics) by subjecting homogenates of frontal cortex to a stepwise fractionation process that extracted material into ‘soluble’, vesicular-peripheral membrane (‘vesicular’), membrane-bound (‘membrane’) and ‘insoluble’ classifications [19]. These solutions were then assayed for total K and Rb content using inductively coupled plasma-mass spectrometry (ICP-MS; see Materials and Methods). In AD frontal cortex significant decreases in both K and Rb levels were apparent (Fig. 1a-b). The degree of change increased in magnitude from the ‘soluble’ to ‘insoluble’ fractions. As expected, a high degree of correlation between tissue K and Rb levels were observed regardless of diagnosis (*r* = 0.862 (HC); *r* = 0.738 (AD); both *p* < 0.001; Fig. 1c-d). However, the slope measured by linear regression was significantly decreased in the AD brains (63.7 % of HC; *F* = 5.41, *p* < 0.05). A receiver operating characteristic (ROC) curve of both K and Rb levels in the total homogenate (which showed the largest difference between clinical classifications) found that Rb performed better at predicting AD, with an area under the curve of 0.815 versus 0.754 for K (both *p* < 0.01; Fig. 1e-f).

**Table 1:** Subject demographics for *post mortem* brain samples

	Healthy controls (*n* = 30)	Alzheimer’s disease (*n* = 30)	*p* value
Age (years)	76.8 (7.6)	78.3 (9.2)	0.56
Females (%)	33.3	26.7	0.59
% APOE ε4 carriers	13.3	75.8	<0.001
*Post mortem* interval (hours)	38.4 (14.3)	33.9 (21.9)	0.38

Mean (standard deviation); *p*-value calculated using a two-tailed Student’s *t*-test

### Serum levels of K and Rb are altered in Alzheimer’s disease

**Fig. 2:**
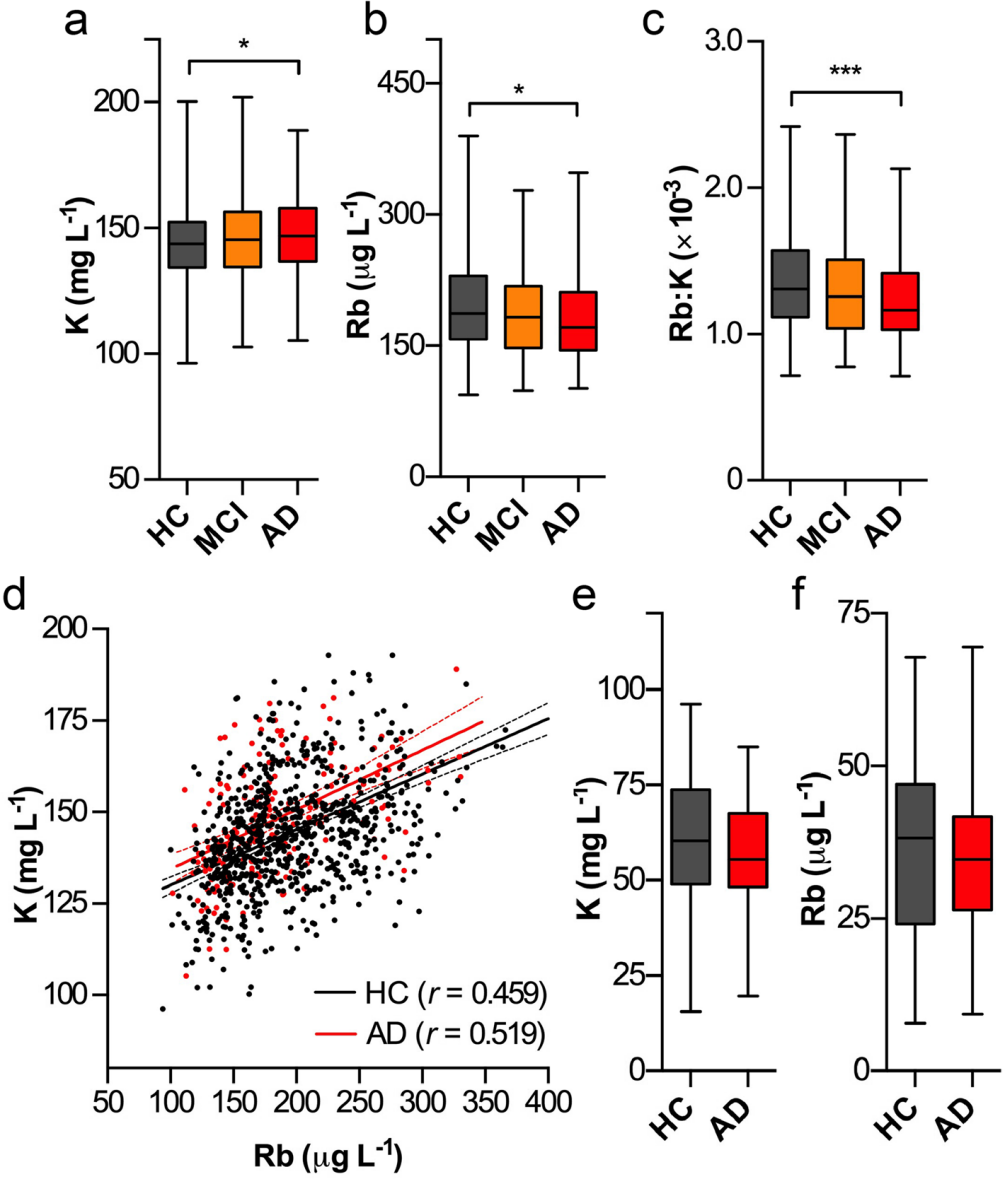
K levels were significantly increased in AD serum (**a**; one-way ANOVA; * *p* < 0.05), whilst Rb levels were conversely decreased in AD (**b**; * *p* < 0.05); the latter mirroring our observation in brain homogenates. The ratio of Rb to K was significantly (*** *p* < 0.001) decreased in AD (**c**), though Rb and K levels were less well correlated in serum (**d**). In a subset (*n* = 30 per group) of erythrocytes from HC and AD groups, there was no significance difference in metal concentration (**e**, **f**). Boxes depict 25^th^ and 75^th^ percentiles and mean; error bars represent minimum to maximum values

To investigate whether the observed changes in K and Rb levels within brain homogenates are reflected in the periphery, we examined serum K and Rb concentrations in the entire baseline AIBL cohort, including the mildly cognitively impaired group (*n*_*total*_ = 1077; *n*_*HC*_ = 778; *n*_*MCI*_ = 128; *n*_*AD*_ = 171; Table 2; see Ellis et al. [18] for cohort full demographics). Significant differences were observed for both analytes between HC and AD groups, but these shifts did not reflect the magnitude or direction of those observed in the brain. There was a 2.6 % increase in serum K levels in the AD subjects (Fig. 2a), with a small (5.4 %) decrease in serum Rb (Fig. 2b). There was a highly significant (*p* < 0.001) decrease in the ratio of Rb to K in the AD group (Fig. 2c), though ROC analysis revealed changes in all three measures were less robust (though still statistically significant) indicators of AD than total Rb and K in brain homogenates (AUC_*K*_ = 0.569; AUC_*Rb*_ = 0.575; AUC_*Rb:K*_ = 0.6115; Additional file 1: Figure S1). K and Rb levels correlated in both groups, although again to a much lesser extent than in brain homogenates, and the slopes of both lines did not differ (*F* = 0.21; *p* = 0.65; Fig. 2d).

**Table 2:** Subject demographics of the baseline AIBL cohort used for serum K and Rb analysis

	Healthy controls (*n* = 778)	Mild cognitive impairment (*n* = 128)	Alzheimer’s disease (*n* = 171)	*p*-value
Age (years)	70.6 (7)	76.2 (7.6)	78.8 (8.6)	<0.001
Females (%)	57.6	56.3	62.0	0.34
% APOE ε4 carriers	27.2	49.2	62.4	<0.001
CDR SOB	0.0 (0.1)	1.2 (0.8)	5.8 (2.9)	<0.001
MMSE	28.9 (1.2)	26.2 (2.7)	18.9 (5.3)	<0.001

CDR SOB is the Clinical Dementia Rating Scale Sum of Boxes score used to stage dementia. MMSE is the mini-mental state examination questionnaire score. *p-*value calculated using a one-way ANOVA or chi-squared test for percentages

### Rb levels are decreased in platelets, but not erythrocytes or CSF

**Fig. 3:**
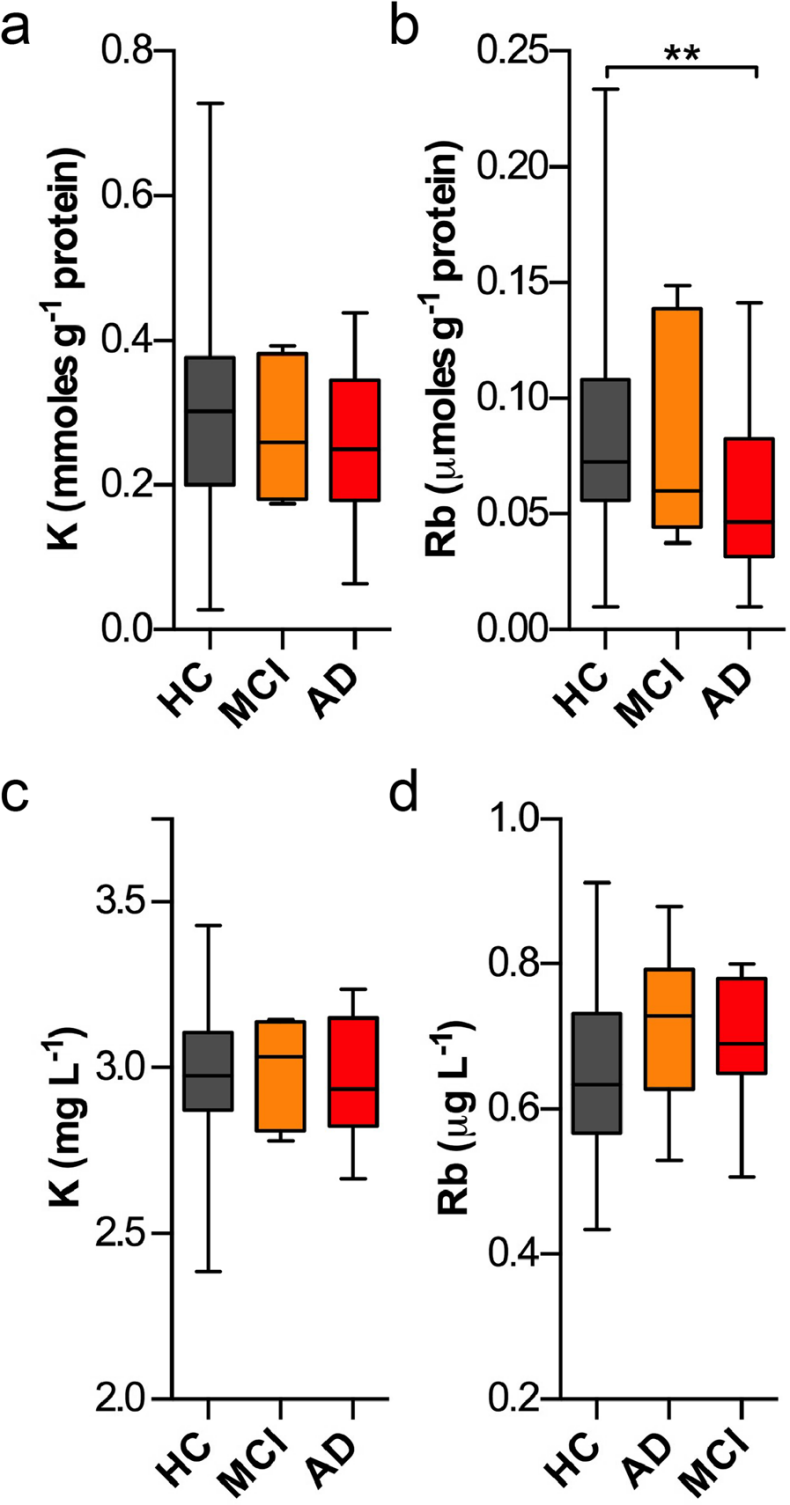
K and Rb levels in platelets and CSF. **a**, **b** In platelets no significant changes were observed for K, though Rb was significantly decreased (*p* < 0.01; one-way ANOVA) in AD subjects compared to controls. **c**, **d** K and Rb levels in CSF remained unchanged. Boxes depict 25^th^ and 75^th^ percentiles and mean; error bars represent minimum to maximum values

We analyzed a subset of AD and HC erythrocytes for Rb and K concentrations (*n* = 40 per classification) and found no significant difference between groups (*p* = 0.62; Fig. 2e, f). K levels in a subset of HC, AD and MCI platelets (*n*_*HC*_ = 50, *n*_*AD*_ = 41, *n*_*MCI*_ *=* 7; Table 3) were not different between groups (Fig. 3a). Rb levels in AD platelets were significantly decreased compared to the HC group (one-way ANOVA; *p* < 0.05; Fig. 3b; Table 4). This decrease in Rb levels in platelets showed limited diagnostic potential when examined using an ROC curve (Additional file 1: Figure S2). In cerebrospinal fluid (CSF; *n*_*HC*_ = 36, *n*_*AD*_ = 9, *n*_*MCI*_ *=* 7; Table 5) K and Rb levels were unaltered according to clinical classification (Fig. 3c, d).

**Table 3:** Subject demographics for erythrocytes

	Healthy controls (*n* = 40)	Alzheimer’s disease (*n* = 40)	*p*-value
Age (years)	82.0 (8.0)	82.0 (8.0)	0.97
Females (%)	52.5	50.0	0.96
% APOE ε4 carriers	37.5	60.0	0.05

*p*-value calculated using a two-tailed Student’s *t*-test

**Table 4:** Subject demographics for platelets

	Healthy controls (*n* = 50)	Mild cognitive impairment (*n* = 7)	Alzheimer’s disease (*n* = 41)	*p*-value
Age (years)	73.8 (6.3)	71.6 (4.8)	80.8 (7.8)	<0.001
Females (%)	60	14.3	75.6	<0.01
% APOE ε4 carriers	20	28.6	75.6	<0.001

*p-*value calculated using a one-way ANOVA or chi-squared test for percentages

**Table 5:** Subject demographics for CSF

	Healthy controls (*n* = 36)	Mild cognitive impairment (*n* = 7)	Alzheimer’s disease (*n* = 9)	*p*-value
Age (years)	71.8 (5.6)	68.3 (4.5)	71.3 (7.1)	0.40
Females (%)	58.0	57.1	33.3	0.41
% APOE ε4 carriers	19	0	44.4	0.20

*p-*value calculated using a one-way ANOVA or chi squared test for percentages

## Discussion

Our results expand upon a previous smaller study that reported decreased Rb levels in the AD brain [23]; we found here that Rb levels are decreased in interstitial/cytosolic, vesicular, membrane-bound and otherwise insoluble fractions, and that these changes correlate with and reflect decreased K levels in the same isolates. Both K and Rb levels have been shown to be decreased in homogenates from a range of brain regions that show progressive neurodegeneration in AD [24]. We found no compelling evidence that K and Rb levels external to the brain (i.e., blood products and CSF) have diagnostic potential in AD research.

Our data suggests that the observed effects of impaired alkali metal metabolism in the AD brain, such as altered Na^+^/K^+^-ATPase activity manifest as a decrease in total brain Rb and K levels, as opposed to being restricted to a specific fraction. We found that the decrease was of a higher magnitude (c.a. 50 %) within cellular membranes (i.e., the ‘membrane’ and ‘insoluble’ fractions). It is unclear as to whether a reduction in K (and, by nature of its ability to exchange, Rb) is a cause or effect of the disease process, though a number of genes associated with K transport and flux have been identified as showing decreased transcription levels in the AD brain [25]. In vitro studies of mixed cortical cultures have shown that Aβ induces K^+^ efflux via enhancing the delayed rectifier K^+^ current *I*_*K*_ [26], potentially through the formation of transient ion channels in bilayer membranes [27].

Importantly, this disruption of cellular metabolism in AD likely commences well before the appearance of histopathological features and the onset of clinical symptoms. Genome-wide transcriptomic analysis of AD brains identified as many as 70 % of genes encoding subunits of the mitochondrial electron transport chain were expressed at lower levels in regions of the brain most affected by AD when compared to controls [28]. Neural network analysis of ‘seed genes’ (the principal genes from which regulatory pathways are grown [29]) identified those encoding the master energy regulator AMP-activated protein kinase as being dysregulated in AD [30].

The effects of impaired energy metabolism appear to primarily result in a loss of membrane integrity through its negative impact on active transport mechanisms that regulate resting membrane potential. As the major consumer of energy in the cell—maintaining resting potential and action potentials accounts for 20–50 % of energy use in neurons in the cortex [31]—the action of Na^+^/K^+^-ATPase is easily compromised by energy deficits. In the AD brain, Na^+^/K^+^-ATPase activity is depressed [32], with a reduction in α3 subunit mRNA observed, a process that occurs prior to the formation of Aβ plaques and is rapidly accelerated in the disease [33], further supporting the hypothesis that an energy crisis is inexorably linked to the molecular basis of the disease.

These findings may have diagnostic potential in the clinical setting. Rubidium-82 has been extensively used in positron emission tomography (PET) as a diagnostic marker for brain tumors [34], with increased uptake of the Rb-82 tracer indicative of a loss of blood-brain barrier (BBB) integrity. Permeability of the BBB is thought to be a feature of both normal aging and AD [35], though both increased serum K concentrations in AD and a failure to identify a change in CSF K or Rb levels tends to suggest that BBB permeability is not directly responsible for the decrease in akali metal levels in the AD brain. Further, a recent study using animal models of AD, including the lipopolysaccharide (LPS) model of induced inflammation, PS2-APP and human tau expressing transgenic mice lines, *APOE* knockout and *APOE4* knock-in mice showed no evidence of BBB permeability compared to both wild type and a positive multiple sclerosis model control using multiple assays, including Rb-86 radiotracer experiments [36], questioning the long-held belief that BBB disruption is characteristic of AD.

Receiver-operating characteristic curve analysis of brain Rb levels (Fig. 2f) shows that changes in levels of this metal is a strong predictor of AD, therefore Rubidium-82/86 PET imaging could be used to identify early-stage AD. Considering that alkali metal homeostasis is disrupted via decreased Na^+^/K^+^-ATPase activity prior to Aβ plaque formation, this approach may have greater preclinical value than the contemporary method employing Pittsburgh compound-B to assess brain amyloid burden by PET [37]. However, if the BBB integrity is maintained in AD, delivery of the imaging agent presents a challenge to use as a preclinical diagnostic tool. An intact BBB still demonstrates a small degree (2.1 %) of Rb uptake, though this is ten-fold less than typically observed in cases of brain tumors and associated BBB disruption [38]. Hyperosmolar BBB disruption via injection of 25 % mannitol to the carotid artery has been shown to induce a temporary window for ^82^Rb loading into the baboon brain [39], though the safety of this approach in humans remains untested beyond patients with pre-existing brain tumors. A non-invasive scanning ultrasound approach to temporarily open the BBB in the APP23 transgenic AD mouse model was shown to be effective in clearing accumulated Aβ [40], and a number of clinical trials testing the safety and efficacy of this approach, predominantly for drug delivery, are currently ongoing [41]. Development of a suitable BBB-permeable vehicle that releases Rb into the brain is an intriguing line of enquiry.

An alternative means to monitor brain Rb levels without the need to deliver an exogenous tracer uses the paramagnetic properties of the naturally occurring ^87^Rb radioisotope, which accounts for approximately 28 % of Rb in the natural environment. *T*_*1*_ and *T*_*2*_ relaxation times are well suited to magnetic resonance imaging using specialized coils, and this approach has been used to quantitatively assess K (using ^87^Rb as a proxy) in the brains of ischemic rats [42, 43]. This approach uses chronic Rb loading via drinking water in the weeks preceding MRI scanning, which could be employed in a diagnostic workflow for AD, though the use of high field strength (e.g., 7 T) MRI systems may have the capacity to assess endogenous ^87^Rb with sufficient sensitivity.

## Conclusions

We have shown that Rb and K levels are consistently decreased across all cellular components in the AD frontal cortex, and altered to a lesser extent in the periphery. As Rb is readily exchangeable with K, we hypothesize that this decrease is indicative of dysfunctional Na^+^/K^+^-ATPase activity, a pathological feature of AD that is representative of an internal energy crisis and precedes the formation of proteinaceous inclusions and neuron loss in the disease. Rubidium levels in the brain performed well in predicating AD, and may represent a new avenue of early diagnosis using existing in vivo imaging techniques, including PET and MRI.

## Additional file

Additional file 1: ROC analysis of Rb & K levels in plasma (Figure S1) and platelets (Figure S2). (DOCX 1297 kb)
