# Supplementary material for: Rubidium and potassium levels are altered in Alzheimer’s disease brain and blood but not in cerebrospinal fluid
Source: Acta Neuropathol Commun. 2016 Nov 14;4:119. doi: 10.1186/s40478-016-0390-8 (PMC5109650; doi:10.1186/s40478-016-0390-8)
Supplement: Additional file 1: — ROC analysis of Rb & K levels in plasma (Figure S1) and platelets (Figure S2). (DOCX 1297 kb) [file 40478_2016_390_MOESM1_ESM.docx]

**Supplementary information for:**

**Rubidium and potassium levels are altered in Alzheimer’s disease brain and blood but not in cerebrospinal fluid**

Blaine R. Roberts^1*^, James D. Doecke^2,3,4^, Alan Rembach^1#^, L. Fernanda Yévenes^1^, Christopher J. Fowler^1^, Catriona A. McLean^1,5,6^, Monica Lind^1^, Irene Volitakis^1^, Colin L. Masters^1^, Ashley I. Bush^1^, Dominic J. Hare^1,7^ and the AIBL research group^8^.

*^1^ The Florey Institute of Neuroscience and Mental Health, The University of Melbourne, Parkville, Victoria, Australia*

*^2^ Cooperative Research Centre for Mental Health, Parkville, Victoria, Australia.*

*^3^ The Australian e-Health Research Centre, Herston, Queensland, Australia*

*^4^ CSIRO Preventative Health Flagship, Molecular Science and Engineering, Parkville, Victoria, Australia*

*^5^ Department of Anatomical Pathology, The Alfred Hospital, Melbourne, Victoria, Australia*

*^6^ Department of Medicine, Central Clinical School, Monash University, Clayton, Victoria, Australia*

*^7^ Elemental Bio-imaging Facility, University of Technology, Sydney, Broadway, New South Wales, Australia*

^8^ *https://aibl.csiro.au/about/aibl-research-team*

*^#^ Deceased*

** To whom correspondence should be addressed. Email: blaine.roberts@florey.edu.au*

**
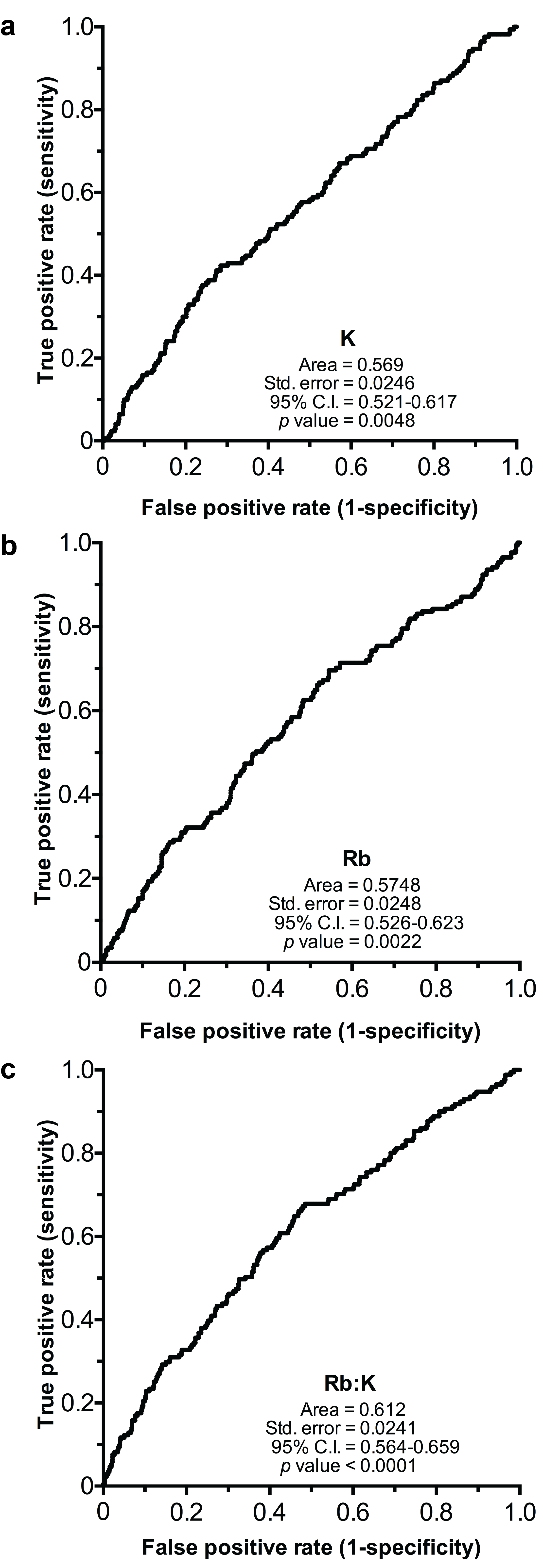
**

**Supplementary Figure 1:** ROC curves for a) K, b) Rb and c) Rb:K in AIBL baseline cohort.


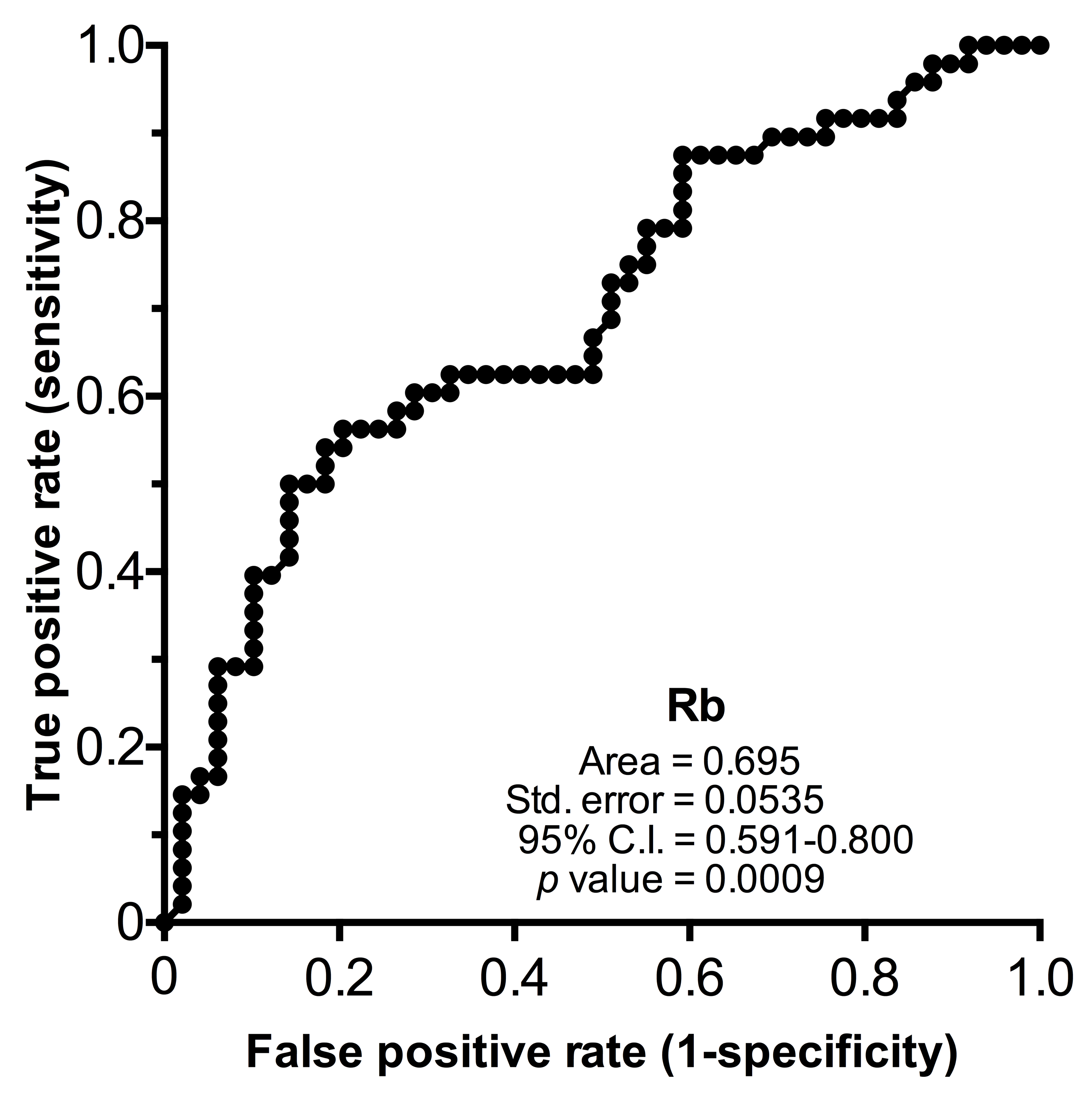


**Supplementary Figure 2:** ROC curve of Rb levels in HC vs AD platelets.
